# Mathematical Model and Solution for Fingering Phenomenon in Double Phase Flow through Homogeneous Porous Media

**DOI:** 10.1155/2013/470174

**Published:** 2013-11-17

**Authors:** Piyush R. Mistry, Vikas H. Pradhan, Khyati R. Desai

**Affiliations:** Department of Applied Mathematics & Humanities, S.V.N.I.T., Surat 395007, India

## Abstract

The present paper analytically discusses the phenomenon of fingering in double phase flow through homogenous porous media by using variational iteration method. Fingering phenomenon is a physical phenomenon which occurs when a fluid contained in a porous medium is displaced by another of lesser viscosity which frequently occurred in problems of petroleum technology. In the current investigation a mathematical model is presented for the fingering phenomenon under certain simplified assumptions. An approximate analytical solution of the governing nonlinear partial differential equation is obtained using variational iteration method with the use of Mathematica software.

## 1. Introduction

Analytical and numerical simulation of the problems arising in oil-water displacement has become a predictive tool in oil industry. In oil recovery process, oil is produced by simple natural decompression without any pumping effort at the wells. This stage is referred to as primary recovery, and it ends when a pressure equilibrium between the oil field and the atmosphere occurs. Primary recovery usually leaves 70%–85% of oil in the reservoir. To recover part of the remaining oil, a fluid (usually water) is injected into some wells (injection wells) while oil is produced through other wells (production wells). This process serves to maintain high reservoir pressure and flow rates. It also displaces some of the oil and pushes it toward the production wells. This stage of oil recovery is called secondary recovery process.

**Figure 1:**
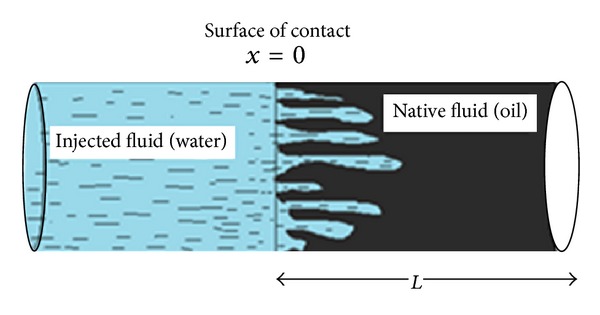
Representation of fingers in a cylindrical piece of homogeneous porous media.

It is a very well-known physical fact that when a fluid having greater viscosity flowing through a porous medium is displaced by another fluid of lesser viscosity then, instead of regular displacement of whole front, protuberance takes place which shoot through the porous medium at a relatively very high speed, and fingers have been developed during this process as shown in Figure 1. This phenomenon is called fingering or instability phenomenon. In the statistical treatment of the fingers only average cross-sectional area occupied by the fingers is considered while the size and shape of the individual fingers are neglected [1]. Many researchers have discussed this phenomenon from various view points. Sheideger and Johnson have discussed the statistical behavior of fingering in homogeneous porous media without capillary pressure [1]. Verma has examined the behavior of fingering in a displacement process through heterogeneous porous media with capillary pressure and pressure dependent phase densities [2]. Mehta has used special relation with capillary pressure and he used singular perturbation technique to find its solution [3]. Verma and Mishra have discussed similarity solution for instability phenomenon in double phase flow through porous media [4]. Pradhan et al. have discussed the solution of instability phenomenon by finite element method [5]. Meher et al. discussed the solution of instability phenomenon arising in double phase flow through porous medium with capillary pressure using Exponential self similar solutions technique [6]. Patel et al. have discussed the power series solution of fingering phenomena in homogeneous porous media [7]. All the above researches have neglected the external sources and sink in the mass conservation equations. In the present study the mathematical model has been presented by considering the mass flow rates of oil and water in the equations of continuity, and the governing nonlinear partial differential equation has been obtained for saturation of injected water.

## 2. Statement of Problem

**Figure 2:**
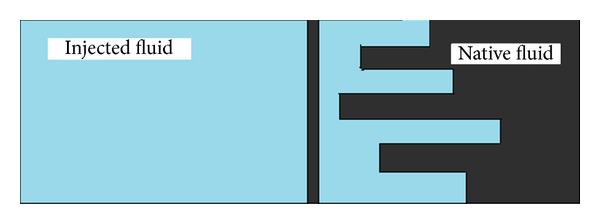
Schematic presentation of fingering (instability) phenomenon.

As shown in Figure 1, a welldeveloped fingers flow is furnished on account of uniform water injection into the oil saturated isotropic, homogeneous porous medium. The schematic presentation of fingers is expressed in Figure 2. Our particular interest in the present investigation is to develop a mathematical model by considering the mass flow rate of oil and water and discuss the fingering phenomenon analytically by using variational iteration method. 

## 3. Mathematical Formulation

The seepage velocity of water (injected fluid) (*V*_*i*_) and oil (native fluid) (*V*_*n*_) is given by Darcy's law [8]

(1) $$\begin{array}{c} V_{i}=-\frac{k_{i}}{\mu_{i}}K\left(\frac{\partial P_{i}}{\partial x}\right),\\ V_{n}=-\frac{k_{n}}{\mu_{n}}K\left(\frac{\partial P_{n}}{\partial x}\right), \end{array}$$

where *K* is the permeability of the isotropic, homogeneous porous medium, *k*_*i*_ and *k*_*n*_ are the respective relative permeability of water and oil, and *P*_*i*_ and *P*_*n*_ are the respective pressure of water and oil, *μ*_*i*_ and *μ*_*n*_ are the respective viscosity of water and oil.

The equations of continuity of two phases are given as [8]

(2) $$\begin{array}{c} \frac{\partial \left(m\rho_{i}S_{i}\right)}{\partial t}+\frac{\partial \left(\rho_{i}V_{i}\right)}{\partial x}=q_{i},\\ \frac{\partial \left(m\rho_{n}S_{n}\right)}{\partial t}+\frac{\partial \left(\rho_{n}V_{n}\right)}{\partial x}=q_{n}, \end{array}$$

where *q*_*i*_ and *q*_*n*_ are the constant mass flow rate of water and oil, *ρ*_*i*_ and *ρ*_*n*_ are density of water and oil, *S*_*i*_ and *S*_*n*_ are the saturation of water and oil, respectively, and *m* is the porosity of the medium. 

From the definition of phase saturation [1],

(3) $$\begin{array}{c} S_{i}+S_{n}=1. \end{array}$$

The capillary pressure *P*_*C*_, defined as the pressure discontinuity of the flowing phases across their common interface, is given by

(4) $$\begin{array}{c} P_{C}=P_{n}-P_{i}. \end{array}$$

For definiteness we assume capillary pressure *P*_*C*_ as a linear function of the saturation of water (*S*_*i*_) as

(5) $$\begin{array}{c} P_{C}=-\beta S_{i}, \end{array}$$

where *β* is a positive constant. 

The relative permeability of water and oil is considered from the standard relationship due to Scheidegger and Johnson [1] given by

(6) $$\begin{array}{c} k_{i}=S_{i}, \end{array}$$

(7) $$\begin{array}{c} k_{n}=S_{n}=1-S_{i}. \end{array}$$

The equations of motion for saturation are obtained by substituting the values of (1) in (2), respectively, as

(8) $$\begin{array}{c} \frac{\partial \left(m\rho_{i}S_{i}\right)}{\partial t}=q_{i}+\frac{\partial }{\partial x}\left[K\rho_{i}\frac{k_{i}}{\mu_{i}}\frac{\partial P_{i}}{\partial x}\right], \end{array}$$

(9) $$\begin{array}{c} \frac{\partial \left(m\rho_{n}S_{n}\right)}{\partial t}=q_{n}+\frac{\partial }{\partial x}\left[K\rho_{n}\frac{k_{n}}{\mu_{n}}\frac{\partial P_{n}}{\partial x}\right]. \end{array}$$

Eliminating ∂*P*_*i*_/∂*x* from (4) and (8) we get

(10) ∂(mρiSi)∂t=qi+∂∂x[Kρikiμi(∂Pn∂x−∂PC∂x)].

Combining (9) and (10) and using (3) we get

(11) 0=(qiρi+qnρn) +∂∂x[(Kkiμi+Kknμn)∂Pn∂x−Kkiμi∂PC∂x].

Integrating (11) with respect to *x*,

(12) C1=(qiρi+qnρn)x +(Kkiμi+Kknμn)∂Pn∂x−Kkiμi∂PC∂x,

where *C*_1_ is a constant of integration.

On simplifying,

(13) ∂Pn∂x=C1(K(ki/μi)+K(kn/μn)) +K(ki/μi)(K(ki/μi)+K(kn/μn))∂PC∂x −((qi/ρi)+(qn/ρn))x(K(ki/μi)+K(kn/μn)).

Substituting the value of (13) in (10),

(14) ∂(mSi)∂t=qiρi+∂∂x[Kkiμi(C1(K(ki/μi)+K(kn/μn))+K(ki/μi)(K(ki/μi)+K(kn/μn))∂PC∂x−((qi/ρi)+(qn/ρn))x(K(ki/μi)+K(kn/μn))−∂PC∂x)].

Expressing *P*_*n*_ as $P_{n}=\overset{-}{P}+\left(1/2\right)P_{C}$, where $\overset{-}{P}=\left(P_{i}+P_{n}\right)/2$ is a constant mean pressure, we have

(15) $$\begin{array}{c} \frac{\partial P_{n}}{\partial x}=\frac{1}{2}\frac{\partial P_{C}}{\partial x}. \end{array}$$

Thus from (15) and (12) we get

(16) C1=(qiρi+qnρn)x +(Kkiμi+Kknμn)12∂PC∂x−Kkiμi∂PC∂x.

Substituting the value of *C*_1_ in (14) and on simplification we have

(17) $$\begin{array}{c} \frac{\partial \left(mS_{i}\right)}{\partial t}=\frac{q_{i}}{\rho_{i}}+\frac{\partial }{\partial x}\left[-\frac{Kk_{i}}{2\mu_{i}}\frac{\partial P_{C}}{\partial x}\right]. \end{array}$$

Using (6) and (5) in (17) and after some simplification, we get

(18) $$\begin{array}{c} m\frac{\partial \left(S_{i}\right)}{\partial t}=\frac{q_{i}}{\rho_{i}}+\left(\frac{K\beta }{2\mu_{i}}\right)\frac{\partial }{\partial x}\left[S_{i}\frac{\partial S_{i}}{\partial x}\right] \end{array}$$

or

(19) $$\begin{array}{c} \frac{\partial S_{i}}{\partial t}=\frac{q_{i}}{m\rho_{i}}+\left(\frac{K\beta }{2m\mu_{i}}\right)\frac{\partial }{\partial x}\left[S_{i}\frac{\partial S_{i}}{\partial x}\right], \end{array}$$

where porosity *m* and permeability *K* are treated as constant for isotropic, homogeneous porous medium. 

Considering the dimensionless variables,

(20) $$\begin{array}{c} X=\frac{x}{L},\;\;T=\frac{K\beta }{2\mu_{i}mL^{2}}t, \end{array}$$

in (19), we get

(21) $$\begin{array}{c} \frac{\partial S_{i}}{\partial T}=A+\frac{\partial }{\partial X}\left[S_{i}\frac{\partial S_{i}}{\partial X}\right], \end{array}$$

where *A* = 2*μ*_*i*_ *L* ^2^ *q*_*i*_/*Kβρ*_*i*_.

In order to solve (21) completely the following specific initial and boundary conditions are considered:

(22) $$\begin{array}{c} S_{i}\left(X,0\right)=f\left(X\right),\\ S_{i}\left(0,T\right)=f_{1}\left(T\right),\\ S_{i}\left(L,T\right)=f_{2}\left(T\right). \end{array}$$

## 4. Solution of Problem

Following the variational iteration method [9–11], we obtain the following iteration formula for (21):

(23) Sik+1(X,T)=Sik(X,T) +∫0T[(−1)(∂Sik∂τ−∂∂X[Sik∂Sik∂X]−A)]dτ.

Define the operator *O*[*S*_*i*_*k*__] as

(24) O[Sik]=(−1)∫0T[(∂Sik∂τ−∂∂X[Sik∂Sik∂X]−A)]dτ.

Define the components *v*_*k*_, *k* = 0,1, 2,…, as

(25) $$\begin{array}{c} v_{0}=f\left(X\right)=0.01X^{2},\\ v_{1}=O\left[v_{0}\right],\\ v_{2}=O\left[v_{0}+v_{1}\right],\\ \vdots \\ v_{k+1}=O\left[v_{0}+v_{1}+\cdots +v_{k}\right]. \end{array}$$

Here the initial approximation *v*_0_ is assumed from the initial condition where the function *f*(*X*) is considered to be in parabolic nature. Pradhan et al. [5] have discussed the fingering phenomenon numerically by assuming *f*(*X*) to be a linear function of space variable.

Using (24) and (25) we get the following iterations with the help of Mathematica software:

(26) v1=−∫0T[(∂v0∂τ−A−∂∂X[v0∂v0∂X])]dτ,v1=T(0.68+0.0006X2),v2=−∫0T(∂{v0+v1}∂τ−A−∂∂X({v0+v1}∂{v0+v1}∂X))dτ,v2=−2.71051×10−20TX2 +T3(0.000272+7.2×10−7X2) +T2(0.0068+0.000036X2),v3=−∫0T(∂{v0+v1+v2}∂τ−A−∂∂X({v0+v1+v2}∂{v0+v1+v2}∂X))dτ,v3=T7(5.59543×10−11+4.44343×10−13X2)  +T6(4.896×10−9+5.184×10−11X2)  +T5(3.5904×10−7+2.592×10−9X2)  +T4(0.00001564+8.64×10−8X2)  +T3(0.000045333+0.00000144X2),v4=−∫0T(∂{v0+v1+v2+v3}∂τ−A−∂∂X({v0+v1+v2+v3}∂{v0+v1+v2+v3}∂X))dτ,v4=T15(3.31505×10−24+7.89762×10−26X2) +T14(7.25168×10−22+1.97441×10−23X2) +T13(8.59045×10−20+2.30347×10−21X2) +T12(7.18117×10−18+1.72761×10−19X2) +T11(4.41144×10−16+9.59781×10−18X2) +T10(2.07243×10−14+4.22303×10−16X2) +T9(8.47521×10−13+1.52854×10−14X2) +T8(3.26248×10−11+4.73225×10−13X2) +T7(1.047744×10−9+1.24416×10−11X2) +T6(2.77168×10−8+2.592×10−10X2) +T5(4.6512×10−7+4.1472×10−9X2) +T4(2.2667×10−7+4.32×10−8X2).

Further approximations can be similarly obtained. Considering the first four approximations, the resulting approximate analytical solution is given by

(27) Si(X,T)=v0+v1+v2+v3+v4=0.01X2 +T15(3.31505×10−24+7.89762×10−26X2) +T14(7.25168×10−22     +1.974405746938776×10−23X2) +T13(8.590445×10−20+2.30347×10−21X2) +T12(7.18117×10−18+1.72761×10−19X2) +T11(4.41144×10−16+9.59781×10−18X2) +T10(2.07243×10−14+4.22303×10−16X2) +T9(8.47521×10−13+1.52854×10−14X2) +T8(3.26248×10−11+4.73225×10−13X2) +T7(1.1037×10−9+1.28859×10−11X2) +T6(3.26128×10−8+3.1104×10−10X2) +T5(8.2416×10−7+6.7392×10−9X2) +T4(0.00001587+1.296×10−7X2) +T3(0.00031733+0.00000216X2) +T2(0.0068+0.000036X2) +T(0.68+0.0006X2).

### 4.1. Convergent Analysis

**Theorem 1**

Let

(28) $$\begin{array}{c} A\left[u\right]=\overset{t}{\underset{0}{\int }}\left\{\begin{array}{c} \frac{{\left(-1\right)}^{m}}{\left(m-1\right)!}{\left(\tau -t\right)}^{m-1}\\ \left[\left({Lu}_{k}\left(\tau \right)+{Nu}_{k}\left(\tau \right)-g\left(\tau \right)\right)\right] \end{array}\right\}d\tau \end{array}$$

be an operator from Hilbert space H to H. The series solution *u*(*t*) = ∑_*k*=0_^*∞*^ *v*_*k*_ converges if ∃0 < *γ* < 1 such that ||*v*_*k*+1_|| ≤ *γ*||*v*_*k*_||  ∀ *k* ∈ *N* ∪ {0} [10], where

(29) $$\begin{array}{c} v_{0}=u_{0},\\ v_{1}=A\left[v_{0}\right],\\ v_{2}=A\left[v_{0}+v_{1}\right],\\ \vdots \\ v_{k+1}=A\left[v_{0}+v_{1}+\cdots +v_{k}\right]. \end{array}$$

**Remark 2**

If the first finite *β*_*i*_, *i* = 1,2,…, *l*, are not less than one and *β*_*i*_ ≤ 1 for *i* > *l*, then, of course, the series solution ∑_*k*=0_^*∞*^ *v*_*k*_(*t*) of problem converges. In other words, the finite terms do not affect the convergence of the series solution [10]:

(30)   β0=||v1||||v0||=68.06, β1=||v2||||v1||=0.0104<1, β2=||v3||||v2||=0.0088<1, β3=||v4||||v3||=0.0122<1, β4=||v5||||v4||=0.0190<1 ⋮

Based on the above theorem the approximate analytical solution given by (27) is convergent.

## 5. Numerical and Graphical Presentation of Solution

**Figure 3:**
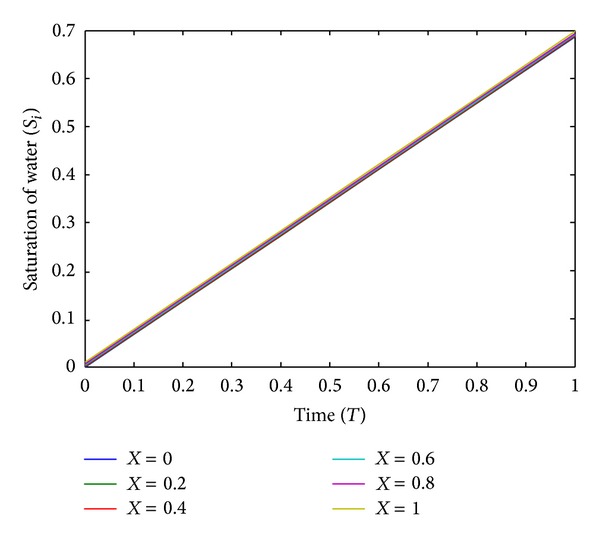
The plot of time (*T*) versus saturation of water (*S*_*i*_) for different values of distance (*X*).

**Figure 4:**
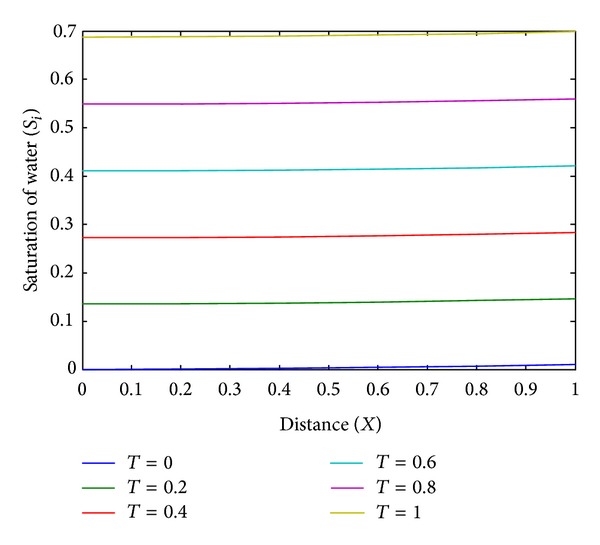
The plot of distance (*X*) versus saturation of water (*S*_*i*_) for different values of time (*T*).

The numerical values of the saturation of water *S*_*i*_(*X*, *T*) are shown in Table 1 for different values of time and distance. The graphical representation of the same has been shown in Figures 3 and 4. From Figures 3 and 4, it is observed that saturation of injected water increases with the space variable *X* and time variable *T*. This resembles well with the physical phenomenon of the problem.

## 6. Conclusion

In the present investigation the phenomenon of fingering has been analytically discussed by considering the mass flow rate of injected water to determine the saturation of injected water for different values of time and distance. It is concluded that by considering the mass flow rate of oil and water, the saturation of injected water advances faster in comparison with the saturation of injected water neglecting the mass flow rate. The values of parameters used in present investigation are shown in Table 2; however the parameters *m* and *K* can be assumed as the function of space variable in the case of anisotropic, heterogeneous porous medium, and the relative permeabilities *k*_*i*_ and *k*_*n*_ are assumed as function of saturation under the equilibrium condition. These relative permeabilities can also be assumed as a function of effective saturation under the nonequilibrium effects. The capillary pressure *P*_*C*_ has been assumed to depend only on the saturation of the wetting phase (water); this capillary pressure can also depend on the surface tension, porosity, permeability, and the contact angle with the rock surface of the wetting phase which in turn depends on the temperature and fluid composition; with such assumption on capillary pressure the parameters can also be included to study its effect in future. Darcy law is considered in two-phase system without the gravitational forces; the differential form of the Darcy law can be extended to three-phase system with and without gravitational forces. The present mathematical model for one-dimensional flow can also be extended to two-dimensional, three-dimensional flows for isotropic, homogeneous and anisotropic, heterogeneous porous media.

In the present study the mass conservation equation and Darcy's law are considered for isothermal flows where the effect of temperature to the system is neglected; however, the mathematical model can be developed for nonisothermal flows. Analytical methods are the most widely used classical reservoir engineering methods in the petroleum industry in predicting petroleum reservoir performance. We concluded that the present variational iteration method used for finding the approximate analytical solution was found to be easy, accurate, and efficient in comparison with other analytical methods.

## Figures and Tables

**Table 1:** Numerical values of saturation of water at different values of time and distance.

*X*	*T*					
	*S*_*i*_ *T* = 0	*S*_*i*_ *T* = 0.2	*S*_*i*_ *T* = 0.4	*S*_*i*_ *T* = 0.6	*S*_*i*_ *T* = 0.8	*S*_*i*_ *T* = 1
*X* = 0	0	0.136275	0.273109	0.410519	0.548521	0.687134
*X* = 0.2	0.0004	0.136679	0.273519	0.410934	0.548941	0.687560
*X* = 0.4	0.0016	0.137894	0.274748	0.412178	0.550202	0.688836
*X* = 0.6	0.0036	0.139918	0.276797	0.414253	0.552303	0.690964
*X* = 0.8	0.0064	0.142752	0.279666	0.417158	0.555244	0.693943
*X* = 1	0.01	0.146396	0.283355	0.420892	0.559025	0.697772

**Table 2:** Values of different parameters.

Parameter	Value
*μ*_*i*_	0.68 × 10^−3^ Pa sec
*q*_*i*_	0.01 kg/m^3^ sec
*β*	20 k Pa
*ρ*_*i*_	1000 kg/m^3^
*K*	10^−12^ m^2^
*L*	1 m
*A*	0.6800

## Nomenclature

*V*_*i*_: Seepage velocity of injected fluid (meter/second)
*V*_*n*_: Seepage velocity of native fluid (meter/second)
*K*: Permeability of homogeneous porous medium (meter^2^)
*k*_*i*_: Relative permeability of injected fluid (dimensionless)
*k*_*n*_: Relative permeability of native fluid (dimensionless)
*μ*_*i*_: Viscosity of injected fluid (pascal second)
*μ*_*n*_: Viscosity of native fluid (pascal second)
*ρ*_*i*_: Density of injected fluid (kg/meter^3^)
*ρ*_*n*_: Density of native fluid (kg/meter^3^)
*q*_*i*_: Mass flow rate of water (kg/(second·meter^3^))
*q*_*n*_: Mass flow rate of oil (kg/(second·meter^3^))
*P*_*i*_: Pressure of injected fluid (pascal)
*P*_*n*_: Pressure of native fluid (pascal)
*m*: Porosity of homogeneous porous medium (dimensionless)
*β*: Capillary pressure coefficient (pascal)
*S*_*i*_: Saturation of water (dimensionless)
*x*: Linear coordinate for distance (meter)
*t*: Linear coordinate for time (second)
*X*: Linear coordinate for distance (dimensionless)
*T*: Linear coordinate for time (dimensionless)
*L*: Length of porous medium (meter)
*P*_*C*_: Capillary pressure (pascal).
